# Spatiotemporal Distribution Characteristic and Influencing Factors of African Swine Fever Outbreaks (2018/8–2019/12) in China

**DOI:** 10.1155/vmi/9954801

**Published:** 2025-10-06

**Authors:** Juan Li, Bingxin Nie, Shubo Li, Junhui Zhang, Lu Gao

**Affiliations:** ^1^School of Computer Science and Technology (School of Artificial Intelligence), Zhejiang Sci-Tech University, Hangzhou, China; ^2^Liaoning Center for Animal Disease Control and Prevention, Shenyang, China; ^3^School of Animal Science and Technology (School of Animal Medicine), Huazhong Agricultural University, Wuhan, China; ^4^China Animal Health and Epidemiology Center, Qingdao, China

**Keywords:** African swine fever (ASF), Bayesian spatiotemporal model, Gross domestic product (GDP), influencing factors, prevention and control, relative risk (RR), veterinary resources

## Abstract

African swine fever (ASF), a highly lethal viral disease with no effective vaccines or treatments, poses a significant threat to the global pig industry. Since its first report in China in August 2018, it has spread rapidly, severely impacting China's pig industry. This study developed a Bayesian spatiotemporal model to explore ASF's spatiotemporal patterns, assess relative risk (RR), and identify key factors, aiming to inform targeted prevention strategies. Data (disease-related deaths, pig inventory, GDP, temperature, and 6 other factors) were collected from 31 mainland Chinese provinces from August 2018 to December 2019. The INLA algorithm estimated parameters, with the optimal model selected via DIC and WAIC. Multicollinearity was addressed using VIF and Spearman's correlation coefficient. Univariate and multivariate models quantified factor effects, with risk classified by natural breaks. Significant spatiotemporal patterns emerged: high-risk clusters in Liaoning, Heilongjiang, and Beijing, lower risk in Yunnan and Chongqing. Economic factors and veterinary resources were crucial: GDP per capita correlated positively (RR = 1.8814, 95% CI: 1.1264, 3.1362), while veterinarian numbers correlated inversely (RR = 0.7233, 95% CI: 0.4776, 0.9637). This study clarifies ASF dynamics and influencing factors in China, highlighting the need to strengthen veterinary services and balance economic development with biosecurity, offering a global reference for infectious disease management.

## 1. Introduction

African swine fever (ASF), an acute, hemorrhagic, and highly contagious disease of pigs, is caused by the ASF virus (ASFV), posing a significant challenge for pig production globally [1]. ASFV has the capacity to infect pigs of all ages, characterized by rapid transmission and a case fatality rate up to 100% [2]. The World Organization for Animal Health (WOAH, formerly OIE) has documented that from January 1, 2022, to February 28, 2025, ASF has been reported in 64 countries across 5 global regions, affecting over 953,000 domestic pigs and more than 33,700 wild boars, and leading to over 2,067,000 animal losses, where Europe has been the most severely impacted, followed by Asia and Africa [3]. These outbreaks not only directly endanger the health of pigs but also inflict significant economic losses upon affected nations. In Africa, for instance, the economic cost of ASF was estimated at US$151,3340 in Benin between 2014 and 2018 [4]; a 2001 epidemic in Nigeria, driven by 91% mortality across 306 farms, resulted in losses of US$941,492 [5]; and an outbreak among 219 households in Tanzania translated to US$41,065 in losses [6]. Outside historically endemic regions, ASF devastated swine production in China, tripling the price of live finishers from approximately 13 to 38 yuan/kg [7], with losses exceeding US$100 billion in 2018 alone [8]. In Vietnam, 20% of pigs died or were culled within the first five months of the outbreak, with 2019 economic impacts ranging from US$880 million to US$4.4 billion [9]. In India, direct losses from animal deaths between April 2020 and June 2021 reached US$37.32 million [10]. Additionally, Russia suffered direct economic losses exceeding 2 billion rubles (approximately 3.2 billion USD) during the 2008–2013 epidemic, with indirect economic losses estimated to be in the range of 20–30 billion rubles [11]. It is estimated that if ASF were to break out in the United States, it could result in a loss of 16.5 billion US dollars in the first year [12]. Recognizing the gravity of this threat, the WOAH has designated it as a legally reportable animal disease, while China has classified it as a priority animal disease for prevention [13]. However, the absence of effective vaccines or drug treatments underscores the urgency of alternative control measures. Culling infected pigs and ensuring robust biosecurity remain the primary means of containing the epidemic [14]. Therefore, the prevention and control of ASF, which stands as the paramount threat to global pig health, necessitates immediate and concerted efforts.

Within this global context, China, as one of the world's largest pig producers and consumers, has faced its own unique set of struggles since the virus first emerged within its borders. The country's experience with ASF not only reflects the broader global trends in disease spread and impact but also presents distinct complexities shaped by its specific agricultural landscape and socioeconomic context. On August 3, 2018, the first case of ASF was diagnosed in Shenyang City, Liaoning Province, China. Subsequently, ASF spread rapidly, affecting 31 provinces, municipalities, and autonomous regions across mainland China. This resulted in a considerable number of pigs infected and culled, causing a catastrophic impact on China's pig farming industry and trade [15, 16]. In response to the severe epidemic, the Chinese government promptly instituted a comprehensive set of preventive and control measures, achieving remarkable successes [17]. However, the complete eradication of ASFV remains a challenge, with periodic resurgences, especially with the emergence of new mutated strains, increasing the urgency of intensified control efforts. China's vast geography, diverse pig breeding systems, and business models, uneven epidemic prevention and control capabilities, as well as regional economic disparities, compound the challenges of combating the epidemic. Furthermore, staffing shortages within epidemic prevention departments, coupled with a complex and dynamic climatic landscape, facilitate the persistence and spread of ASFV. To effectively tackle the ASF epidemic, it is imperative to adopt scientific and rational approaches to delve into the spatial and temporal distribution patterns, epidemiological trends, and key influencing factors of ASF. By assessing the relative risk (RR) levels across different regions, we can formulate targeted preventive and control strategies, thereby safeguarding the security and stability of the pig industry.

Bayesian spatiotemporal model has emerged as a pivotal analytical tool for studying data characterized by complex spatial and temporal dynamics, particularly in the field of infectious disease transmission [18]. By assuming all unknown parameters are random variables and setting reasonable prior distributions, the Bayesian spatiotemporal model enhances the accuracy of risk assessment and addresses heterogeneity within complex spatiotemporal structures by harnessing the interconnectedness of neighboring spatiotemporal data [19]. Unlike traditional statistical methods, the Bayesian spatiotemporal model boasts the capability to concurrently analyze the temporal, spatial, and correlative aspects of diseases, thereby affording a more comprehensive assessment and prediction capability [20]. The model has been extensively applied in the field of space epidemiology to investigate a range of diseases, including malaria [21], hand, foot and mouth disease [22], Ebola [23], schistosomiasis [24], renal syndrome hemorrhagic fever [25], Clonorchis sinensis [26], brucellosis [27], and hydatid disease [28]. These studies have not only unraveled the underlying the law of governing disease transmission and spatiotemporal distribution, but also conducted thorough evaluations of various influencing factors. Consequently, they have provided invaluable insights that have guided the development of prevention and control strategies as well as public health policies, underscoring the model's transformative impact on our understanding and management of infectious diseases.

Despite the widespread utilization of Bayesian spatiotemporal modeling in numerous disease studies, the current research landscape falls short in comprehensively elucidating the spatiotemporal propagation pattern of ASF and its underlying influencing factors within China. Therefore, the objective of this study is to construct a Bayesian spatiotemporal model, tailored specially to China's ASF scenario and related data, to examine the spatial and temporal dynamics of ASF propagation across mainland China from August 2018 to December 2019. Additionally, this study will analyze the impacts of socioeconomic and natural factors that significantly shape the trajectory of ASF's spread. By quantitatively assessing the contribution of each factor to the ASF incidence risk, we will clarify the RR level of each region and identify the most influencing factors. By enhancing our understanding of the spatiotemporal dynamics of ASF and its underlying drivers, we can advance towards implementing proactive and targeted strategies for prevention and control.

## 2. Materials and Methods

### 2.1. Data Resources

This study is organized around four primary data categories:

1. ASF epidemic data. We have compiled and summarized monthly data on pig deaths and culling due to disease in all 31 provincial administrative units in mainland China from August 2018 to December 2019 (see Table 1). The data for each outbreak, reported specifically on farms where cases have been identified, originate from the China Animal Health and Epidemiology Center. These data adhere to a rigorous collection process and standards outlined in the study by Gao et al. [29].
2. Pig production and management statistics. This dataset encompasses a wide range of variables essential to pig farming and veterinary services. These variables cover year-end live pig inventories, slaughter numbers, slaughterhouse counts, veterinary stations, veterinarian personnel, and farm numbers for each provincial entity. The data were extracted from the China Animal Husbandry and Veterinary Yearbook [30, 31]. To address the challenge of monthly live pig inventory unavailability, we have adopted a weight ratio allocation method based on provincial year-end stocks and national monthly live pig stocks from August 2018 to December 2019 [7], to estimate the monthly live pig stock. With the assumption that the proportion of monthly pig inventory to the annual total in each province remains consistent with that of the whole country, the specific expression of this method is as follows:
  (1) $$\begin{array}{c} S_{P_{ijk}}=S_{p_{ij}}\times \frac{S_{N_{jk}}}{\sum_{k=1}^{12}S_{N_{jk}}}, \end{array}$$
where *S*_*P*_*ijk*__ is represents the live pig stock for the *k*-th month of the *j*-th year in the *i*-th province, *S*_*p*_*ij*__ denotes the live pig stock for the *j*-th year in the *i*-th province, and *S*_*N*_*k*__ is the national live pig stock for the *k*-th month of the *j*-th year.
3. Annual gross domestic product (GDP) data. Providing insights into economic conditions, this section features GDP per capita and total regional GDP from the China Statistical Yearbook [32, 33]. These figures reflect the average income levels of residents across the study period. To ensure consistency in the analysis, we have directly applied the 2018 and 2019 GDP per capita and regional total GDP data for each month, disregarding monthly GDP fluctuations.
4. Meteorological data. Obtained primarily from the China Meteorological Data Service Centre's ground meteorological stations [34], this dataset comprises daily 6-h precipitation and air temperature readings. By calculating averages, we have derived monthly metrics of temperature and precipitation for each provincial unit, offering valuable insights into climatic conditions during the study period.

### 2.2. Research Methods

#### 2.2.1. Bayesian Spatiotemporal Model and Solution Methods

In previous studies that employed Bayesian spatiotemporal model to simulate the infectious diseases, incidence metrics or incidence count statistics have been frequently utilized to evaluate disease transmission patterns [35]. For ASF-specific research, studies examining the spatiotemporal variation of China's epidemic and its transmission risk factors have predominantly relied on case counts [36, 37] and incidence rates [38]. However, such metrics face critical limitations in the ASF context, as demonstrated in research on high-consequence animal diseases: delayed reporting and undercounting of subclinical infections, common issues in livestock epidemics [39], which introduce significant bias into incidence-based estimates. In addition, given ASF's unique characteristics, including its rapid spread, short disease course, and near 100% mortality in infected herds [2], accurate case counting remains challenging, which limits the reliability of relying solely on case counts to assess the epidemic's spread. Notably, ASF outbreaks typically result in massive mortality or mandatory culling of affected herds, making live pig losses (encompassing both disease-related deaths and preventive culling) a more comprehensive and measurable indicator. Compared to incidence-based metrics, live pig losses better capture the full extent of epidemic impact in ASF scenarios. Therefore, we have opted to adopt a modified approach, using the metric of live pig losses (both disease-related deaths and culling) as an alternative to the traditional morbidity count in our model, aiming to enhance the accuracy and reliability of ASF spread assessments.

The ratio of monthly live pig losses to the total live pig inventory in each province is low, indicating that live pig losses account for a relatively small percentage of the total. Based on this observation, we assume that the amount of live pig losses, denoted by *y*_*it*_, due to ASF epidemic in month *t*(*t* = 1, 2,…, 17) in the *i*-th (*i* = 1, 2,…, 31) province follows a Poisson distribution. Then, we construct a model as follows:

(2) $$\begin{array}{c} y_{it}∼\text{Poisson}\left(E_{it}\theta_{it}\right), \end{array}$$

where *θ*_*it*_ is the RR of ASF incidence in *i*-th province during month *t*, which serves as an indicator of whether the RR value is higher (*θ*_*it*_ > 1) or lower (*θ*_*it*_ < 1) than the national average RR.*E*_*it*_ is the expected number of live pig losses with the expression (*E*_*it*_ = *S*_*P*_*it*__ × (∑_*i*=1_^31^*y*_*it*_/∑_*i*=1_^31^*S*_*P*_*it*__)), where *S*_*P*_*it*__ represents the live pig stock for the *t*-th month in the *i*-th province. The Bayesian spatiotemporal model developed in this study is formulated as follows:

(3) $$\begin{array}{c} {\log\left(\theta_{it}\right)=\beta_{0}+u_{i}+v}_{i}+\varphi_{t}+\gamma_{t}+\delta_{it}+\sum \beta_{k}X_{itk}. \end{array}$$

In the expression (3), *β*_0_ is the intercept, capturing the average log RR across the entire study area; *u*_*i*_ and *v*_*i*_ denote the spatial structure and spatial nonstructure effects, respectively. The spatial structure effect, reflecting the correlation between neighboring regions, follows a conditional autoregressive (CAR) prior distribution [40], which is represented by an *N* × *N* adjacency matrix (*N* is the number of regions), where w_*ij*_ = 1 if regions (*i*) and (*j*) are adjacent and 0 otherwise. The spatial nonstructure effect represents the heterogeneity within the region; *φ*_*t*_ and *γ*_*t*_ denote the temporal nonstructure and temporal structure effect, measured in monthly units, respectively. The temporal nonstructure effect indicates temporal heterogeneity, while temporal structure effect reflects monthly trends, with its prior distribution obeying a first-order dynamic random walk model (RW(1)), assuming randomness in the differences between adjacent time points and a degree of temporal continuity; *δ*_*it*_ denotes the spatiotemporal interaction effect, which represents the combined effect of time and space on the research variable y_it_. This interaction term is specified as shown in Table 2 [41]; *X*_*itk*_ denotes the *k*-th influencing factor associated with RR with *β*_*k*_ being its corresponding regression coefficient. Except for the temporal and spatial structure effects, all remaining hyperparameters are assumed to follow a Gaussian distribution with a mean of 0 and a variance of *σ*^2^. All variables are summarized in Table 3.

The current approaches for solving Bayesian spatiotemporal models are Markov chain Monte Carlo (MCMC) methodology introduced by Gelfand et al. [42] and the integrated nested Laplace approximation (INLA) technique proposed by Rue et al. [43]. In this study, we will use the INLA method, primarily due to its significantly reduced computation time compared to MCMC. Second, the INLA method is particularly well-suited for models with sparse structures, such as generalized linear mixed models and spatial models [44]. For these types of models, the INLA method offers more refined approximation results and demonstrates exceptional proficiency in handling large-scale datasets.

#### 2.2.2. Evaluation Criteria

The primary evaluation criteria for Bayesian spatiotemporal model are the deviance information criterion (DIC) introduced by Spiegelhalter et al. [45] and the Watanabe–Akaike information criterion (WAIC) proposed by Watanabe [46].

DIC serves as a pivotal model selection criterion, adept at comparing Bayesian statistical models by balancing the model's goodness of fit with its complexity. This ensures that the chosen model describes the data without succumbing to overcomplexity. DIC achieves this equilibrium by combining the average log likelihood of the model with a measure of its complexity, which is formulated as follows:

(4) $$\begin{array}{c} \text{DIC}=D\left(\overset{¯}{\theta }\right)+2p_{D}=\overset{¯}{D}+p_{D}, \end{array}$$

where $D\left(\overset{¯}{\theta }\right)$ quantifies the deviation from the posterior mean, serving as a proxy for the model's goodness of fit, while $\overset{¯}{D}$ denotes the posterior mean of this deviation. *p*_*D*_ represents the effective number of parameters employed by the model, to assess its complexity. A higher *p*_*D*_ value indicates an increased model complexity.

WAIC, widely known as a robust Bayesian information criterion [47], is rooted in the harmonious blend of pointwise predictive density and model complexity. This integration balances a model's ability to conform to the data with its capacity for generalization, while accommodating Bayesian model structures. The formula for WAIC is given by

(5) $$\begin{array}{c} \text{WAIC}=-2\times lppd+2\times p_{\text{WAIC}}, \end{array}$$

where *lppd*, or the log pointwise predictive density, assesses the model's log likelihood on a given dataset by computing and summing the log predictive density for each individual data point. Conversely, *p*_WAIC_ evaluates the model's complexity and parameter freedom degrees by computing the variance of the likelihood function. Similar to DIC, WAIC also assesses the model's fit and complexity, but with an added precision in estimating the model's generalization error—its predictive prowess on unseen data. This fine-grained assessment of individual data points' impact on model fit mitigates potential imbalances encountered with criteria like DIC. However, it is crucial to note that WAIC, like any tool, has its limitations. In specific Bayesian model scenarios, particularly those involving intricate structures or a high proportion of missing data, WAIC may be prone to substantial biases. Recognizing the strengths and limitations of both DIC and WAIC, this study adopts both as evaluation metrics for model quality. A lower value for either DIC or WAIC signifies a more optimal model fit.

#### 2.2.3. Seasonality Analysis

Seasonal-trend decomposition using Loess (STL) is a time series decomposition method proposed by Cleveland et al. [48]. This method can decompose the time series *Y*(*t*) into three components: the trend term *T*(*t*) (reflecting the long-term change direction of the time series), the seasonal term *S*(*t*) (embodying periodic fluctuations), and the residual term *R*(*t*) (the remaining part after removing the trend and seasonality, which usually contains noise or random fluctuations).

Given that the seasonal cycle involved in the study is 12 months, we set the parameter “period” to 12; all other parameters adopt default values: both “seasonal_deg” (the polynomial order of seasonal smoothing) and “trend_deg” (the polynomial order of trend smoothing) are 1, and “robust” (whether to enable robust processing to correct outliers) is set to the default state.

The specific decomposition steps are as follows: First, the Loess method is used to fit the trend component *T*(*t*) of the original sequence, and the detrended component *Y*(*t*) − *T*(*t*) is calculated; then, the detrended data are split into multiple sub-sequences according to the cycle (e.g., 12 months), and the seasonal component *S*(*t*) is fitted for each sub-sequence by Loess; next, the deseasonalized sequence *Y*(*t*) − *S*(*t*) is calculated, and the Loess is used again to refit the trend component *T*(*t*); finally, the residual term is obtained by *R*(*t*) = *Y*(*t*) − *T*(*t*) − *S*(*t*). After completing the decomposition, we visually presented the original sequence *Y*(*t*), the trend term *T*(*t*), the seasonal term *S*(*t*), and the residual term *R*(*t*).

#### 2.2.4. Analysis of Influencing Factors

The epidemiological dynamics of ASF in China are complex, influenced by a web of spatial and temporal correlations with biological, social, and environmental factors. To comprehend this intricate progress, we collected data on 10 potentially relevant factors, including live pig inventory, number of veterinarians, GDP, and average temperature, and others, across 31 provinces, municipalities, and autonomous regions of China.

To investigate the factors influencing the spread of ASF in China, it is essential to select explanatory variables closely linked to ASF spread and develop a multifactor Bayesian spatiotemporal model based on these factors. A critical step involves screening potential variables to address the issue of multicollinearity, which can undermine the robustness of model coefficient estimates and diminish their explanatory power. To tackle this challenge, we adopt the variance inflation factor (VIF) as a diagnostic tool to identify and exclude variables exhibiting high multicollinearity [49]. A VIF value exceeding 10 signals significant collinearity, necessitating the exclusion of such variables to enhance model accuracy. Nevertheless, recognizing the limitations of VIF in detecting nonlinear relationships, we supplement our analysis with the Spearman correlation coefficient method [50]. This approach enables us to identify variables with strong nonlinear correlations (i.e., correlation coefficients |ρ| > 0.5), which may indicate covariance that could be overlooked by VIF-based analysis alone [51].

Furthermore, we conducted a single-factor Bayesian spatiotemporal model analysis to identify the factors that exert a significant impact on the RR of ASF incidence. This analysis was marked by excluding any variable with 95% credible interval (CI) of RR that did not include 1, indicating statistical significance. Ultimately, factors with low collinearity and significant impact were integrated into a multifactor Bayesian spatiotemporal model. This approach enables us to quantitatively assess the individual influence of each identified factor on the risk of ASF transmission, thereby providing robust empirical insights to enhance our understanding of the disease and guide strategies for its containment and control.

### 2.3. Software

In this study, we employed two primary software applications to facilitate data processing and analysis. Specifically: (1) Microsoft Excel served as the cornerstone for data recording, ensuring accurate documentation, and its organizational capabilities streamlined the data for preliminary statistical analysis, providing us with an initial understanding of the dataset. (2) R 4.2.3 was employed to delve deeper into the correlations between the data, performing advanced analysis. The key packages included INLA 23.04.24, spdep 1.2–8, sf 1.0–16, SpatialEpi 1.2.8, and several others. This involved conducting collinearity analysis based on the VIF to address potential multicollinearity issues, exploring the correlations between the influencing factors to gain valuable insights, and constructing a Bayesian spatial–temporal model to capture the dynamic and spatial aspects of the data. The results were subsequently visualized for enhanced comprehension and interpretation.

## 3. Result

### 3.1. Descriptive Statistical Analysis

According to the statistics of the epidemiological data obtained as of December 2019, mainland China has recorded a cumulative total of 177 outbreaks involving over 20,000 infected live pigs, resulting in the culling of at least 1.22 million pigs. Figures 2 and 3 depict the monthly trends and spatial distribution of these outbreaks and subsequent pig losses, respectively.

As Figure 2 demonstrates, the number of ASF outbreaks and associated live pig losses exhibited a distinct pattern of gradual increase followed by decline, devoid of any discernible seasonal fluctuations. Notably, most outbreaks clustered between September and December 2018 and in April 2019, with a secondary peak in April 2019. Following this, the outbreak frequency diminished significantly, with no more than five incidents recorded per month post-April 2019. The epidemic peaked in October 2018, inflicting catastrophic losses of over 350,000 pigs, highlighting the gravity of the ASF threat.

Figure 3(a) illustrates a spatial aggregation of ASF outbreaks, with most regions experiencing fewer than six outbreaks. Notably, the eastern coastal region and northern Shaanxi province stand out as hotspots with 2 to 6 outbreaks each. In contrast, Gansu province and the Inner Mongolia autonomous region exhibited relatively low outbreaks, peaking at six cases. The southwestern region, including Sichuan, Guizhou, and Yunnan provinces, showed a higher incidence rate, with outbreaks ranging from six to 13 cases. Liaoning province endured the most severe outbreaks, with 27 cases of ASF and losses exceeding 270,000 pigs. When considered alongside Figure 3(b), it becomes evident that despite a relatively low number of outbreaks, the eastern coastal regions suffered significant pig losses, exceeding 12,700 heads. A prime example in Fujian Province, which recorded a mere three outbreaks during the study period, yet incurred stagging losses of 67,306 pigs. This underscores the disproportionate impact of ASF on pig populations, even in regions with a seemingly limited outbreak presence.

A comparison of the two subplots in Figure 3 reveals that regions marked by a high prevalence of outbreaks do not invariably exhibit a synchronized increase in pigs' mortality rates. This observation underscores the importance of considering the spatial density of pig breeding in epidemiological analyses of disease outbreaks. It becomes clear that relying solely on a single metric, whether it be the number of outbreaks, fatalities attributed to the epidemic, or the quantity of pigs culled, falls short in comprehensively evaluating the spatial and temporal risks associated with ASF across diverse regions. A multifaceted approach, considering multiple variables and their interactions, is imperative for an accurate and effective assessment of the disease's epidemiology.

### 3.2. Seasonality Analysis

STL decomposition results indicated that the epidemic presented an overall downward trend, while the seasonal components displayed low amplitude and lacked periodic fluctuation patterns that align with seasonal variations (see Figure 4). Further variance analysis of the seasonal components indicated that their contribution to total variance was less than 5%, which was substantially lower than that of the trend components and random fluctuations. Collectively, these findings suggest the absence of significant seasonal effects in the study data.

### 3.3. Spatiotemporal Effects Distribution of ASF RR

In this study, we employed seven Bayesian spatiotemporal interaction models to fit the data, with quantitative indicators DIC and WAIC to evaluate the efficacy if each model's fitting. The specific values of these criteria are detailed in Table 5.

Comparing the DIC values of the different models, we observe that model C has the smallest value, followed by model A. However, an analysis of the WAIC values indicates a different trend, with model A corresponding to the smallest indicator value, whereas model C yields the largest. Adhering to the principle of minimizing evaluation indicators, we carefully consider both criteria. Consequently, model A emerges as the optimal choice, serving as the baseline model for subsequent analyses, further exploring the characteristics of the spatiotemporal distribution and the underlying influencing factors of ASF, which will provide a scientific foundation for the prevention and control strategies against this epidemic.

#### 3.3.1. Spatial Effect Analysis

The study utilized the Bayesian spatiotemporal model, with *RR*_*s* derived from the expression exp(*u*_*i*_ + *v*_*i*_), to assess the spatial effects within *i*-th province. This approach facilitated the determination of the comparative risk between provinces. For visual and quantitative representation of these spatial effects, Figure 5 presents the mean distribution diagram of *RR*_*s*, while Table 6 provides a tabular summary of the spatial effects.

In accordance with the epidemic-level classification outlined in the “Emergency Response Implementation Plan for African Swine Fever Epidemic (2019 Edition),” as issued by the Ministry of Agriculture and Rural Affairs of the People's Republic of China (MARA of PRC) [52], this study adopted the natural point break method to categorize the RR level of ASF into four distinct levels. As shown in Figure 4, variations in the mean *RR*_*s* across provinces underscore spatial heterogeneity. Notably, there exists a spatial clustering of high ASF RR, predominantly in the northeastern and western regions, contrasting with the relatively lower risk observed in central and coastal areas. Key high-risk areas pinpointed include Heilongjiang, Liaoning, Beijing, and Tianjin, which are geographically contiguous and collectively identified as a major zone within the “Work Plan for Zonal Prevention and Control of African Swine Fever and Other Major Animal Epidemics (for Trial Implementation)” [53], specifically the northern region. Furthermore, Hebei, Gansu, and Jilin also exhibit heightened RR levels. Conversely, regions such as Chongqing, Hubei, and Yunnan display low-to-medium ASF RR.

#### 3.3.2. Temporal Effect Analysis

The computation of global temporal effects for Bayesian spatiotemporal model is contingent upon the expression exp(*φ*_*t*_ + *γ*_*t*_).  Figure 6 visually captures the temporal evolution of the RR of outbreaks between August 2018 and December 2019. The findings reveal a distinct pattern, characterized by an initial surge in the RR of the epidemic, followed by a pronounced decline. In particular, the RR is high (*RR*_*t* > 1) until May 2019, mirroring the trends observed in Figure 2. Remarkably, peak RR values were attained in October 2018, December 2018, and April 2019. However, since May 2019, there has been a steady decrease in the RR to levels below unity (*RR*_*t* < 1). This significant shift underscores the remarkable efficacy of the prevention and control measures enacted by MARA of PRC in effectively managing the ASF epidemic.

#### 3.3.3. Spatiotemporal RR Distribution Analysis

To illustrate the rationality of the RR, we will first introduce the definition of the standardized mortality ratio (SMR) and then conduct a comparative analysis. SMR is a key metric for assessing the relationship between observed and expected mortality rates within a specified animal population in epidemiological studies [54]. By dividing the observed deaths by the expected deaths, the SMR provides insights into the RR level in each area. While it shares similarities with the RR in this regard, the SMR faces challenges in accurately portraying potential risk levels, particularly in regions with small breeding sizes or limited outbreak data, due to its inability to fully leverage information from adjacent temporal and spatial contexts. To enhance the visual comprehension of monthly SMR values across regions, we integrated these values with a geographical map, resulting in a SMR risk map as depicted in Figure 7.

This approach facilitates a more nuanced understanding of regional risk patterns. To address the limitations of the SMR, we adopted a model-driven strategy, plotting the regional distribution of RR estimates derived from the Bayesian spatiotemporal model. This approach offers a visualization of the estimated RR for each region, with darker shades representing higher risk levels, as shown in Figure 8, and further animated in the file video_ASFRR18-19.mp4 (Supporting Information). This model-based representation not only complements the SMR analysis but also provides a more comprehensive assessment of epidemic risk.

As illustrated in Figure 7, higher SMR was only shown in the areas where outbreaks occurred from September to December 2018 and in April 2019. Conversely, other areas maintained low-risk levels, exhibiting neither temporal nor spatial trends. For instance, despite an outbreak in Liaoning in August 2018, SMR in Inner Mongolia is shown as zero in Figure 7, even though an ASF outbreak occurred there in September of the same year. Figure 8 reveals a gradual increase in the RR of each region during the peak epidemic period, with a subsequent decline after January 2019. However, there was a temporary resurgence in risk from February to April 2019, followed by another decline and stabilization at a lower level, mirroring the trend observed in Figure 2. Spatially, the distribution of RR demonstrates a notable pattern of regional clustering. The RR fluctuates in tandem with temporal trends in most areas, with notably higher risks in eastern and northern China, gradually tapering off towards the central and southwestern regions. This spatial pattern underscores the complexity and dynamic nature of epidemic risk.

### 3.4. Analysis of Influencing Factors

To identify the significant influencing factors that could contribute to the construction of a multifactor Bayesian spatiotemporal model for the ASF epidemic in China, we conducted a step-by-step analysis of 10 potential factors. This approach aimed to examine each variable and determine its relevance and impact on the epidemic dynamics.

#### 3.4.1. Multicollinearity Analysis

**Figure 1:**
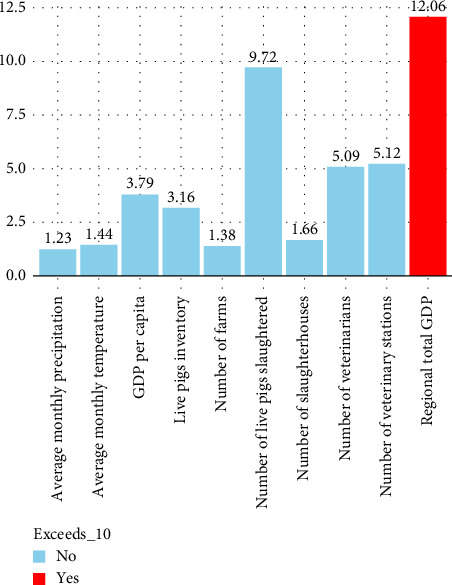
VIF value of influencing factors.

**Figure 2:**
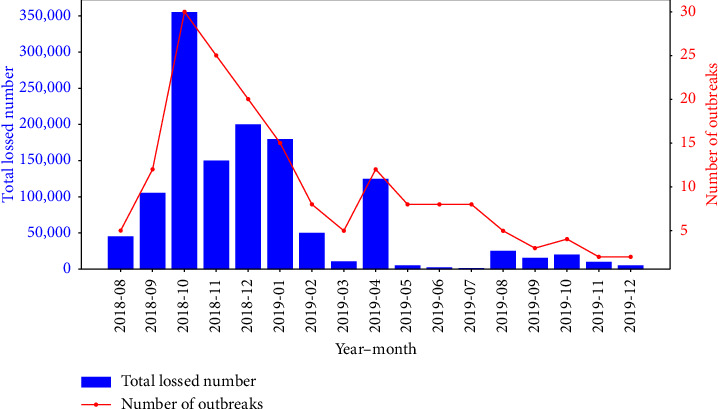
Time series of the number of ASF outbreaks and live pig losses in China, with the number of outbreaks (red line graph, values correspond to the right *Y*-axis) and the number of live pig losses (blue bar graph, values correspond to the left *Y*-axis) (2018.08–2019.12).

**Figure 3:**
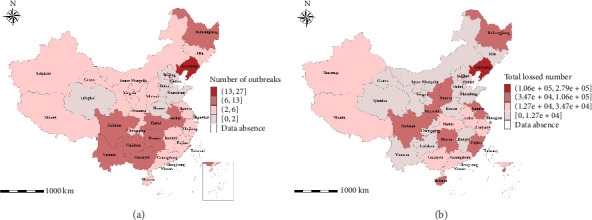
Spatial distribution of the number of ASF outbreaks (a) and live pig losses (b) in China (2018.08–2019.12).

**Figure 4:**
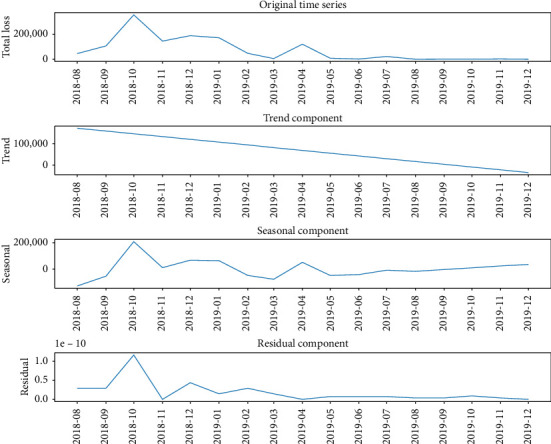
STL decomposition of time series: Live pig losses due to ASF (2018.08–2019.12).

**Figure 5:**
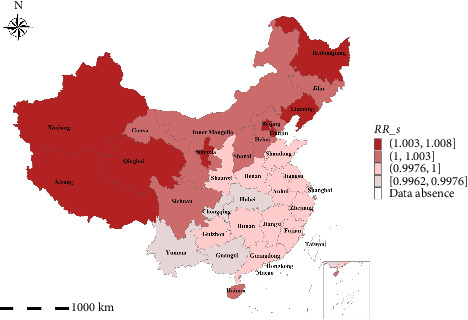
Spatial distribution of mean relative risk (*RR*_*s*) of ASF incidence by region in China.

**Figure 6:**
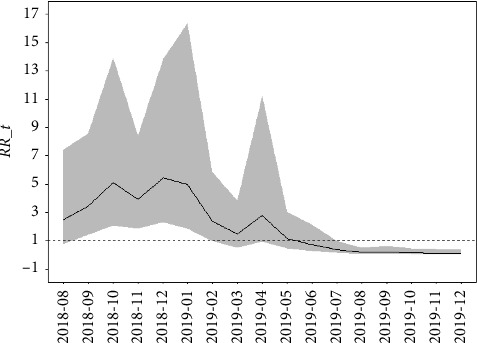
Temporal trends in relative risk (*RR*_*t*) of ASF outbreaks in China (2018-08 to 2019-12), with the shaded regions corresponding to the 95% confidence intervals.

**Figure 7:**
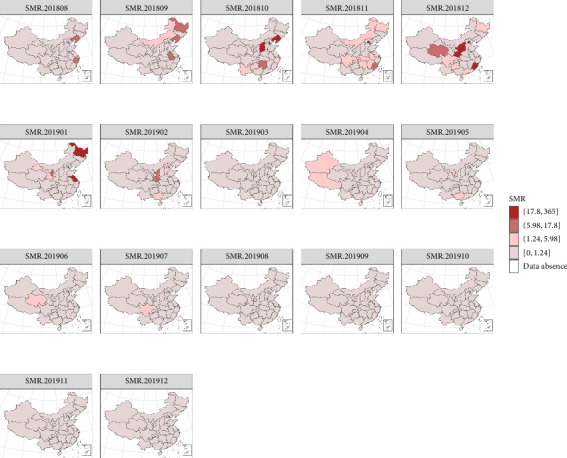
Regional distribution of standardized mortality ratio (SMR) estimates of ASF in China by month (2018.08–2019.12).

**Figure 8:**
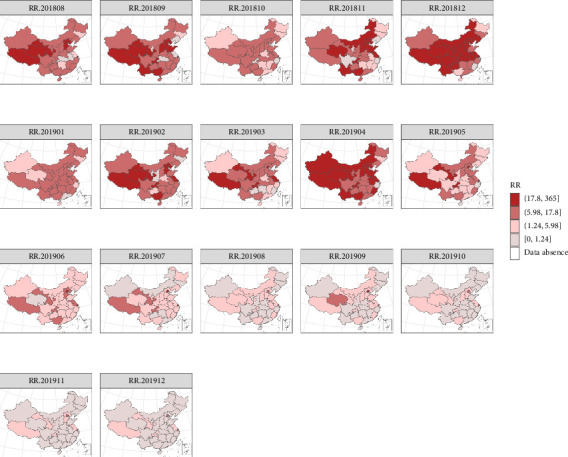
Regional distribution of relative risk (RR) of ASF estimated by Bayesian spatiotemporal model in China by month (2018.08–2019.12).

**Figure 9:**
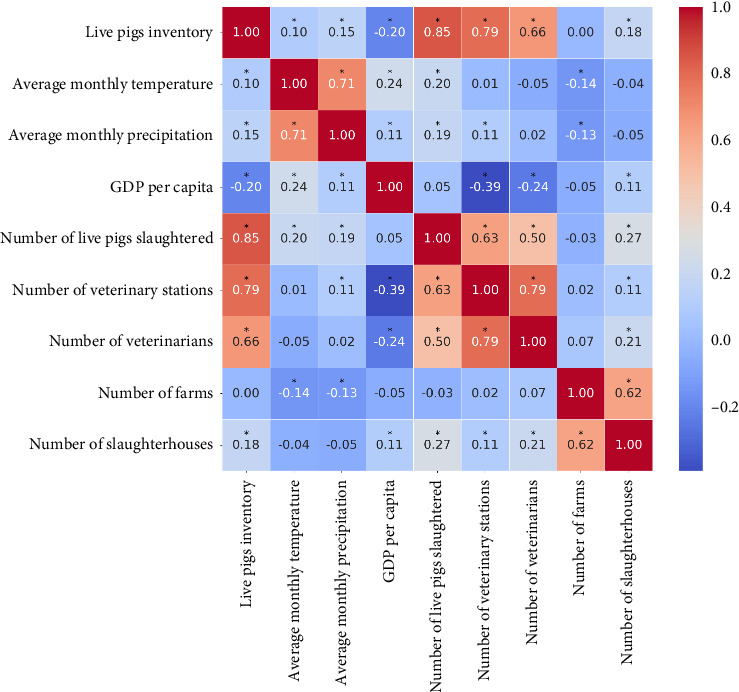
Spearman's correlation coefficient matrix (^∗^ indicates *p* < 0.05).

In this study, to address the potential issue of multicollinearity among the 10 potential influencing factors of the ASF epidemic in China, we computed the VIF for each variable. As depicted in Figure 1, we excluded those factors with VIF values exceeding 10, indicating significant multicollinearity. Notably, regional GDP was found to have a VIF value greater than 10; therefore, it was omitted from further analysis. Subsequently, we reassessed the remaining nine factors using Spearman's correlation coefficient, as shown in Figure 9. Our analysis revealed strong correlations between the number of veterinary stations and several other variables, including the number of veterinarians (*ρ* = 0.79, *p* < 0.05), the number of live pigs slaughtered (*ρ* = 0.63, *p* < 0.05), and the number of live pig inventory (*ρ* = 0.79, *p* < 0.05). Similarly, the number of live pig inventory showed high correlations with the number of live pigs slaughtered (*ρ* = 0.85, *p* < 0.05), veterinary stations (*ρ* = 0.79, *p* < 0.05), and veterinarians (*ρ* = 0.66, *p* < 0.05). Given these robust correlations, we decided to exclude both the number of veterinary stations and the number of live pig inventory from the subsequent single-factor analysis to mitigate potential biases and ensure the validity of our model.

#### 3.4.2. Single-Factor Analysis

Following the multicollinearity analysis, we analyzed the remaining seven potential factors using a single-factor Bayesian spatiotemporal model, with the results presented in Table 4. The results highlighted those three factors—GDP per capita, the number of veterinarians, and the number of live pigs slaughtered—had a significant impact on the RR of ASF incidence (95% CI excluding 1). Among the above-mentioned influencing factors, our findings revealed a positive correlation between GDP per capita and the RR of ASF incidence, suggesting that economic wealth may be associated with an increased likelihood of ASF outbreaks. Conversely, we observed negative correlations for both the number of veterinarians and the number of live pigs slaughtered, indicating that these factors could potentially mitigate the risk of ASF. Based on the results from the single-factor Bayesian spatiotemporal model, we have identified GDP per capita, the number of veterinarians, and the number of live pigs slaughtered as key variables for further analysis within the framework of a multifactor Bayesian spatiotemporal model.

#### 3.4.3. Multifactor Analysis

Table 7 depicts the outcomes of the multifactor Bayesian spatiotemporal analysis, offering a view of the determinants the RR of ASF incidence over the study period. The analysis indicates that the number of live pigs slaughtered did not significantly impact on the RR of ASF incidence, with its 95% CI excluding 1. However, contrasting trends emerged for the other two factors. An upsurge in GDP per capita was positively correlated with an increased risk of ASF incidence, reflected by a RR of 1.8814 (95% CI: 1.1264, 3.1362). Conversely, an increase in the number of veterinarians was inversely associated with the risk, yielding a RR of 0.7233 (95% CI: 0.4776, 0.9637). Figures S1 and S2 in Supporting Information illustrate the spatial distribution of RR for GDP per capita and veterinarian number, respectively. To illustrate these findings in practical terms, consider the following scenarios: if GDP per capita were to rise by 10% from its original base, the RR of ASF incidence would escalate to 1.8814 times the original risk. In contrast, a 10% increase in the number of veterinarians would lead to a decrease in the RR of ASF incidence to 0.7233 times the original level, demonstrating the protective effect of increased veterinary resources.

## 4. Discussion

The statistical analysis revealed a nuanced pattern in the RR of ASF incidence in mainland China during the study period. Initially, there was a noticeable rise in the risk, followed by a decrease and subsequent stabilization, without apparent seasonal variations. Notably, there were brief intervals of heightened risk, specifically during September to December 2018 and April 2019. Conversely, since May 2019, the RR has undergone a marked reduction, strongly correlated with the fact that after the discovery of ASF, the government quickly established a multidimensional and progressive prevention and control system, with various policies and measures forming a synergistic effect, effectively blocking the virus transmission chain [29]. In terms of key policy efforts, starting from October 2018, the government not only explicitly prohibited interprovincial transportation of live pigs but also simultaneously suspended the toll-free policy for live pig transportation [36]. Through dual controls, cross-regional movement of live animals was reduced, cutting off the main path of virus spread at the circulation link. In December of the same year, the prevention and control strategy was further upgraded by implementing comprehensive regionalized prevention and control (dividing the whole country into five regions) and optimizing the form of live pig production and circulation, allowing direct transactions between qualified farms and slaughtering enterprises within the province [53]. This minimized the risk of cross-regional transmission while ensuring pork supply. In addition, the implementation of early basic prevention and control measures laid an important foundation for containing the epidemic. In September 2018, live pig markets in provinces affected by the epidemic were closed, and a nationwide inspection of farms, markets, slaughterhouses, and harmless disposal plants was carried out simultaneously to achieve early detection and disposal of risks. In response to the association between ASF and swill feeding, all provinces across the country completely banned swill feeding [55, 56], eliminating infection hazards at the source of breeding. The issuance of the African Swine Fever Contingency Plan (2019 Version) in January 2019 further standardized the emergency disposal procedures and improved the efficiency of epidemic response [52, 56]. In this process, veterinary experts undoubtedly played a key supporting role in epidemic diagnosis and traceability, guiding the construction of biosecurity in farms, researching and promoting prevention and control technologies, and providing professional suggestions for policy formulation [29, 57].

Compared to SMRs utilized in conventional statistical approaches, the Type *δ*_*it*1_ spatiotemporal interaction model demonstrated its superiority in integrating adjacent spatiotemporal data, thereby enabling a more precise depiction of fluctuations in ASF RR. Furthermore, the study identified geographical clustering of this risk, with pronounced elevations in the northern and western regions and relatively lower levels in the central and southwestern areas, aligning with previous research findings [38]. However, the spatiotemporal dynamics exhibited notable disparities across regions, potentially influenced by a myriad of factors, including geographical location, pig farming intensity, and the effectiveness of epidemic prevention strategies [37, 58, 59]. These findings underscore the complexity and dynamism of ASF risk in China and the importance of tailored interventions for different regions.

Indeed, the risk of ASF incidence is multifaceted and pervasive, even in regions with lower live pig populations like Beijing and Tianjin, necessitating constant vigilance. Statistics showed significant differences in the average values of indicators such as the number of veterinary staff, GDP, slaughter volume, stocking density, number of outbreaks, and number of deaths nationwide [59]. Among them, the average number of veterinary staff is the largest. The number of veterinary staff presents a significant right-skewed distribution (data greater than the average is concentrated on the right), whereas indicators like GDP and the number of outbreaks show the opposite skewness. Our comprehensive analysis delved into 10 potential determinants and pinpointed two key factors: GDP per capita and the number of veterinarians, which significantly shaped the prevalence of ASF outbreaks in China. The correlation between GDP per capita and the RR of ASF incidence was positive, suggesting economic prosperity may inadvertently foster conditions conducive to the spread of the virus. The potential reasons may include: first, GDP per unit area is an excellent indicator reflecting output density and economic development level. In regions with high GDP, the demand for meat products is relatively large, and the road density tends to be high, which increases the connectivity in the pig trade network. According to existing research findings, there is a strong correlation between road density, especially the density of rivers and highways, and ASF outbreaks [58]. These regions usually have the characteristics of more active cross-regional pig circulation, and the increase in trade frequency and scale may increase the risk of virus transmission. Second, in terms of breeding density, due to the developed economy, regions with high GDP are often characterized by large-scale and intensive pig farming, which makes the breeding density relatively high. According to existing research results, there is also a strong correlation between breeding density and ASF outbreaks [58]. While improving efficiency, this high-density breeding also increases the risk of epidemic outbreaks. On the one hand, the living space of pig herds is relatively compact, and once an epidemic occurs, it is easy to spread quickly; on the other hand, high-density breeding has higher requirements for the control of the breeding environment, such as ventilation and sanitation conditions. If the management is poor, it will create conditions for the breeding of epidemics. Conversely, the number of veterinarians emerged as a protective factor, negatively correlated with the risk, highlighting the crucial role of veterinary expertise in mitigating outbreaks. In high-GDP regions like Beijing and Tianjin, where the veterinarian-to-population ratio is relatively low, this imbalance may hinder timely surveillance, precise diagnosis, and effective intervention, thereby elevating the risk of ASF outbreaks. Conversely, provinces like Chongqing, Guangxi, Hubei, and Yunnan, despite their generally lower GDP per capita, were classified as low-risk due to their abundant veterinary resources. This underscores the transformative power of strategic veterinary resource allocation in containing the spread of animal diseases, even in economically less developed areas. Our findings underscore the intricate interplay between economic development and epidemiological dynamics, emphasizing the need for a holistic approach that integrates economic considerations with robust veterinary infrastructure and human resource development. By doing so, we can effectively mitigate the risk of ASF and other animal diseases, safeguarding both public health and economic stability.

Although this study provides a scientific reference for the prevention and control of ASF epidemics, it is important to acknowledge its inherent limitations. Our findings, based solely on our established dataset, lack universal applicability. Notably, data acquisition challenges have precluded the inclusion of all potential factors influencing ASF outbreaks, such as the transportation chain, thereby somewhat restricting the study's depth and breadth. Additionally, the integrity and precision of epidemic data are hampered by issues like underreporting and missed reporting [39, 59], which introduce uncertainty into the study's conclusions. Furthermore, while prevention and control policies significantly influence ASF transmission, quantifying their specific impacts is challenging, making it difficult to incorporate policy effects into our analysis. The ASF control is a vast and systematic endeavor. To ensure that research outcomes can serve this prevention and control effort with greater precision and efficiency, it is imperative for us to adopt a more open and inclusive mindset, fostering deep interdisciplinary and cross-sectoral collaboration and extensive exchanges, and collectively contributing to the establishment of a safe, healthy, and sustainable environment for livestock development.

## 5. Conclusion

Utilizing Bayesian spatiotemporal modeling and rigorous data analysis methodologies (including VIF method, Spearman correlation coefficient, and single-factor analysis), this study investigates the spatiotemporal dynamics of ASF outbreaks in mainland China from August 2018 to December 2019. Key factors with significant impacts on the ASF epidemic were identified while avoiding high multicollinearity. Subsequently, a multifaceted Bayesian spatiotemporal model was proposed to assess each factor's influence on the epidemic's progression. The findings highlight the necessity of region-specific prevention and control strategies tailored to local conditions, such as economic status, population density, and veterinary resource availability. For example, regions with higher per capita GDP should prioritize ASF risk mitigation, while those with limited veterinary resources need capacity building; high-risk areas require strengthened early warning systems and refined response strategies to ensure timely and effective control.

This research offers a scientific foundation and valuable insights for enhancing the prevention and control of ASF. It introduces novel perspectives that will guide future research in this field, emphasizing the importance of a holistic approach that integrates diverse factors to address the complexity of ASF dynamics. By clarifying the spatiotemporal patterns and key drivers of ASF outbreaks, the study contributes to bridging gaps in current understanding, supporting evidence-based decision-making for animal health and public safety, and ultimately helping to establish a robust defense against the devastating impacts of this highly contagious disease.

The current study has certain limitations. First, the scope of influencing factors considered may be incomplete, as the intricate interactions and specific mechanisms driving epidemic spread require deeper exploration. Second, data on live pig and pork transportation networks, though analyzed, may lack comprehensiveness, limiting the accuracy of portraying interregional commodity flows and their impacts. Third, policy impact assessment remains qualitative, and quantitative translation of epidemic policies into measurable data is insufficient, hindering precise evaluation of their effects on the pig industry. Additionally, data integration across sources could be further strengthened to enhance the comprehensiveness, accuracy, and timeliness of the dataset.

To address the identified limitations and advance ASF research, future research should expand the scope to encompass a broader range of influencing factors and delve deeper into their intricate interactions and specific mechanisms driving epidemic spread. This entails several key initiatives: first, deepening the investigation into the transportation network of live pigs and pork by incorporating more extensive data to accurately portray the flow of these commodities across regions and uncover their inherent connections and mutual influences. Second, conducting quantitative policy impact research by translating epidemic policies into quantifiable data and exploring their specific ramifications on the pig industry, which involves building suitable quantitative models, performing comparative analyses of pre- and postpolicy data, and scientifically assessing policy effectiveness. Lastly, strengthening data collection and integration efforts to enhance research accuracy and detail, ensuring data comprehensiveness, accuracy, and timeliness, while also striving to integrate data from various sources into a more comprehensive and systematic dataset. This deeper understanding will empower the development of even more effective prevention and control strategies.

Through ongoing research and practical application, we aim to enhance the comprehensiveness and efficacy of ASF prevention and control measures. Our ultimate goal is to establish a robust defense for animal health and human safety, shielding both from the devastating impacts of this highly contagious disease.

## Figures and Tables

**Table 1:** Thirty-one provincial-level administrative regions in China and their collected number of data points (2018.08–2019.12).

Province	Culled_Number
Anhui	34,510
Beijing	34,860
Chongqing	11,065
Fujian	67,429
Gansu	2575
Guangdong	17,110
Guangxi	27,492
Guizhou	12,758
Hainan	78,698
Hebei	5600
Heilongjiang	104,596
Henan	12,839
Hubei	22,703
Hunan	81,669
Jiangsu	107,610
Jiangxi	4333
Jilin	3140
Liaoning	300,314
Inner Mongolia	8164
Ningxia	3577
Qinghai	1123
Shaanxi	67,030
Shandong	4504
Shanghai	3926
Shanxi	57,804
Sichuan	58,316
Tianjin	18,217
Xinjiang	34,115
Xizang	6763
Yunnan	16,936
Zhejiang	11,027

**Table 2:** Bayesian spatiotemporal model with different spatiotemporal interaction terms.

Expression form	Interaction term of *δ*_*it*_
*δ*_*it*1_	*v*_*i*_ & *φ*_*t*_
*δ*_*it*2_	*v*_*i*_ & *γ*_*t*_
*δ*_*it*3_	*u*_*i*_ & *φ*_*t*_
*δ*_*it*4_	*u*_*i*_ & *γ*_*t*_

**Table 3:** Variables in the model.

Variable	Description	Reference
*u*_*i*_	Spatial structure effect	[35, 40, 41]
*v*_*i*_	Spatial nonstructure effect	[35, 40, 41]
*γ*_*t*_	Temporal structure effect	[35, 40, 41]
*φ*_*t*_	Temporal nonstructure effect	[35, 40, 41]
*δ*_*it*_	Spatiotemporal interaction effect	[35, 40, 41]
*X*_*itk*_	*k*-th influencing factor, refer to Table 4 and Figure 1	—

**Table 4:** Relative risk and 95% credible interval for single-factor model.

Influencing factor	RR	95% CI
Average monthly temperature	0.4661	(0.1334, 1.6821)
Average monthly precipitation	0.8120	(0.4503, 1.4968)
GDP per capita	2.0464	(1.2412, 3.3660)
Number of veterinarians	0.5507	(0.3890, 0.7776)
Number of farms	1.1320	(0.7400, 1.7358)
Number of live pigs slaughtered	0.5024	(0.2952, 0.8535)
Number of slaughterhouses	0.7713	(0.4310, 1.3797)

**Table 5:** Fitting results of Bayesian spatiotemporal models.

Model	Equation	DIC	WAIC
A	log(*θ*_*it*_) = *β*_0_ + *u*_*i*_ + *v*_*i*_ + *φ*_*t*_ + *γ*_*t*_ + *δ*_*it*1_ + ∑*β*_*k*_*X*_*itk*_	1485.30	1459.75
B	log(*θ*_*it*_) = *β*_0_ + *u*_*i*_ + *v*_*i*_ + *φ*_*t*_ + *γ*_*t*_ + *δ*_*it*2_ + ∑*β*_*k*_*X*_*itk*_	1486.06	1461.53
C	log(*θ*_*it*_) = *β*_0_ + *u*_*i*_ + *v*_*i*_ + *φ*_*t*_ + *γ*_*t*_ + *δ*_*it*3_ + ∑*β*_*k*_*X*_*itk*_	1479.19	1527.43
D	log(*θ*_*it*_) = *β*_0_ + *u*_*i*_ + *v*_*i*_ + *φ*_*t*_ + *γ*_*t*_ + *δ*_*it*4_ + ∑*β*_*k*_*X*_*itk*_	1485.78	1461.07
E	log(*θ*_*it*_) = *β*_0_ + *φ*_*t*_ + *γ*_*t*_ + *δ*_*it*1_ + ∑*β*_*k*_*X*_*itk*_	1897.40	2156.36
F	log(*θ*_*it*_) = *β*_0_ + *v*_*i*_ + *φ*_*t*_ + *γ*_*t*_ + *δ*_*it*1_ + ∑*β*_*k*_*X*_*itk*_	1822.58	2118.57
G	log(*θ*_*it*_) = *β*_0_ + *u*_*i*_ + *φ*_*t*_ + *γ*_*t*_ + *δ*_*it*1_ + ∑*β*_*k*_*X*_*itk*_	1772.78	2054.76

**Table 6:** Relative risk (*RR*_*s*) levels for spatial effects by province.

Risk level	*RR_s*	Region
Heavy high risk	(1.003, 1.008]	Beijing, Heilongjiang, Liaoning, Ningxia, Qinghai, Tianjin, Xizang, Xinjiang
High risk	(1, 1.003]	Gansu, Hainan, Hebei, Jilin, Inner Mongolia, Shanxi, Sichuan
Medium risk	(0.9976, 1]	Anhui, Fujian, Guangdong, Guizhou, Henan, Hunan, Jiangsu, Jiangxi, Shandong, Shanghai, Shaanxi, Zhejiang
Low risk	[0.9962, 0.9976]	Chongqing, Guangxi, Hubei, Yunnan

**Table 7:** Relative risk and 95% credible interval for multifactor model.

Influencing factor	RR	95% CI
GDP per capita	1.8814	(1.1264, 3.1362)
Number of veterinarians	0.7233	(0.4776, 0.9637)
Number of live pigs slaughtered	0.6194	(0.3393, 1.1286)

## Data Availability

The data that support the findings of this study are available from the corresponding authors upon reasonable request.

## Supporting Information

Supporting Information 1 Figure S1: Spatial distribution of RR for GDP per capita.

Supporting Information 2 Figure S2: Spatial distribution of RR for number of veterinarians.

Supporting Information 3 Video: ASFRR18-19.
