# Supplementary figures and images for: Spatiotemporal Distribution Characteristic and Influencing Factors of African Swine Fever Outbreaks (2018/8–2019/12) in China
Source: Vet Med Int. 2025 Oct 6;2025:9954801. doi: 10.1155/vmi/9954801 (PMC12517988; doi:10.1155/vmi/9954801)

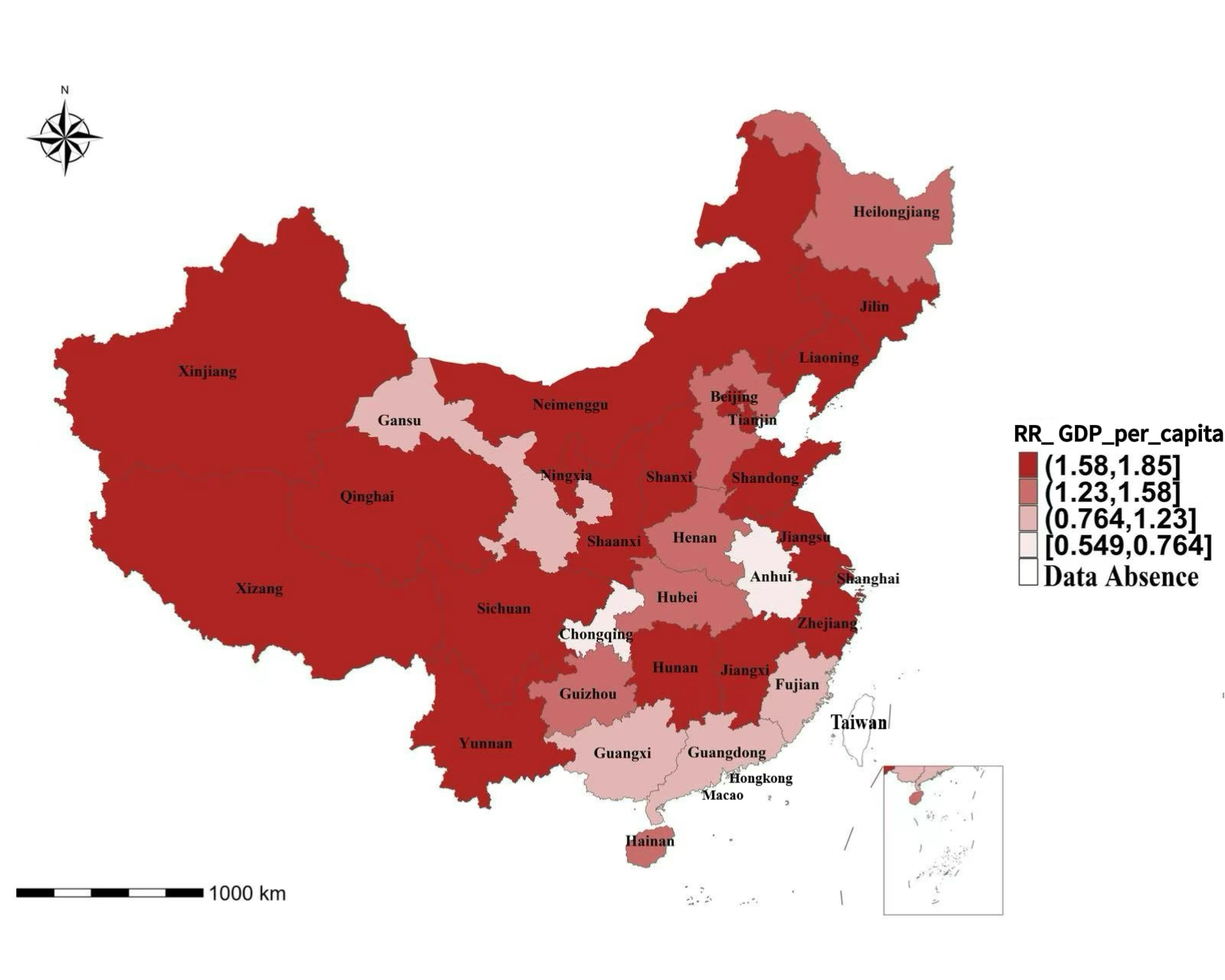

Supplement: Supporting Information 1 — Figure S1: Spatial distribution of RR for GDP per capita. [file 9954801.f1.jpg]

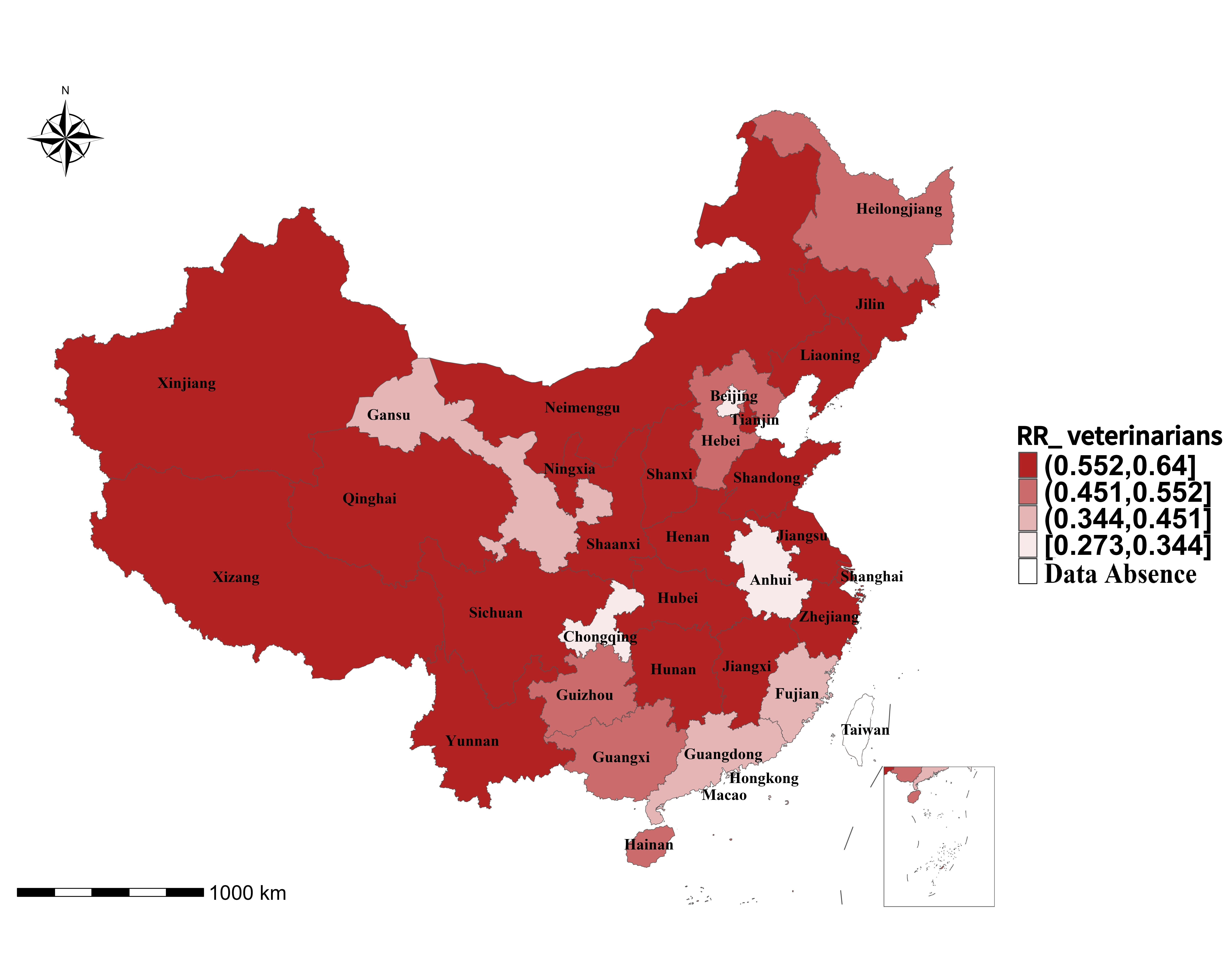

Supplement: Supporting Information 2 — Figure S2: Spatial distribution of RR for number of veterinarians. [file 9954801.f2.jpg]
